# Ticagrelor and Eptifibatide Bolus Versus Ticagrelor and Eptifibatide Bolus With 2‐Hour Infusion in High‐Risk Acute Coronary Syndromes Patients Undergoing Early Percutaneous Coronary Intervention

**DOI:** 10.1161/JAHA.117.005562

**Published:** 2017-06-13

**Authors:** Moazez J. Marian, Oluseun Alli, Firas Al Solaiman, Brigitta C. Brott, Mark Sasse, Tara Leesar, Sumanth D. Prabhu, Massoud A. Leesar

**Affiliations:** ^1^ Division of Cardiology University of Alabama at Birmingham AL

**Keywords:** antiplatelet therapy, eptifibatide, inhibition of platelet aggregation, ticagrelor, Catheter-Based Coronary and Valvular Interventions, Pharmacology, Revascularization

## Abstract

**Background:**

In patients with non‐ST‐segment elevation acute coronary syndromes, inhibition of platelet aggregation (IPA) with a potent P2Y_12_ inhibitor, ticagrelor, was inferior to tirofiban infusion at 2 hours, indicating that glycoprotein IIb/IIIa inhibitors are still needed. Ticagrelor and eptifibatide bolus only may maximally inhibit platelet aggregation and decrease bleeding, but IPA with ticagrelor and eptifibatide bolus versus 2‐hour infusion is unknown.

**Methods and Results:**

A total of 70 P2Y_12_‐naïve patients, with high‐risk non‐ST‐segment elevation acute coronary syndromes, were randomized to ticagrelor and eptifibatide bolus (group 1) versus ticagrelor and eptifibatide bolus with 2‐hour infusion (group 2). Levels of IPA with ADP, thrombin receptor‐activating peptide, collagen, and high on‐treatment platelet reactivity were measured by light transmission aggregometry at baseline and at 2, 6, and 24 hours after percutaneous coronary intervention in both groups. The primary end point, IPA with ADP 20 μmol/L at 2 hours, was 99.59±0.43% in group 1 versus 99.88±1.0% in group 2 (*P*<0.001 for noninferiority). High on‐treatment platelet reactivity with ADP was zero at 2, 6, and 24 hours in both groups. IPA levels with ADP, thrombin receptor‐activating peptide, and collagen were significantly higher at 2 and 6 hours than at 24 hours in both groups. Periprocedural myocardial infarction was not significantly different between the groups. Hemoglobin level was significantly less at 24 hours versus baseline in group 2 (13.35±1.8 versus 12.38±1.8 g/dL, respectively; *P*<0.01).

**Conclusions:**

Ticagrelor and eptifibatide bolus maximally inhibited platelet aggregation at 2 hours, which was associated with no significant hemoglobin drop after percutaneous coronary intervention. This obviates the need for eptifibatide 2‐hour infusion and might decrease bleeding complications.

**Clinical Trial Registration:**

URL: http://www.clinicaltrials.gov. Unique identifier: NCT01919723.

**Clinical Perspective**

**What is New?**

- Ticagrelor and eptifibatide bolus maximally IPA at 2 hours and obviates the need for eptifibatide 2‐hour infusion.
- Platelet reactivity was below the cut point for ischemic risk throughout the 24‐hour period with ticagrelor and eptifibatide bolus.

**What are the Clinical Implications?**

- Current guidelines recommend a glycoprotein inhibitor infusion for 12 to 18 hours in high‐risk ACS patients, which increases the risk of bleeding.
- The combination of ticagrelor and eptifibatide bolus only is likely to reduce the risk of bleeding in high‐risk ACS patients.
- The absence of high platelet reactivity with the use of the combination of ticagrelor and eptifibatide bolus only is likely to reduce event rates.

## Introduction

It has been demonstrated that the level of inhibition of platelet aggregation (IPA) is an independent predictor for the risk of adverse events after percutaneous coronary intervention (PCI).^1^ A recent pharmacodynamic (PD) study^2^ showed that IPA with ticagrelor alone was inferior to tirofiban infusion at 2 hours in patients with non‐ST‐segment elevation acute coronary syndromes (NSTE‐ACS). Furthermore, another recent PD study^3^ showed that the rate of high on‐treatment platelet reactivity (HPR) was still high at 2 hours in patients with unstable angina treated with ticagrelor alone. Likewise, a study showed^4^ that HPR was observed in a significant number of ACS patients (25.2%) who were treated with a loading dose of prasugrel at the time of PCI. The above series indicate that even fast‐acting P2Y_12_ inhibitors, such as ticagrelor, or prasugrel, do not achieve maximal platelet inhibition after 2 hours of loading, the time frame in which patients undergo PCI. Furthermore, the rate of HPR was still high at 2 hours, suggesting that a glycoprotein IIb/IIIa receptor inhibitor (GPI) is needed to achieve maximal platelet inhibition.

Current guidelines^5^ and package labeling recommend a GPI (eg, eptifibatide infusion for 18–24‐hours or tirofiban infusion for 12–18‐hours) in patients with high‐risk NSTE‐ACS undergoing PCI. Despite the well‐established anti‐ischemic effect of GPI, an extended infusion of GPI was associated with increased risk of bleeding and mortality.^6^ On the other hand, a number of series reported that the rate of bleeding significantly decreased with a bolus or short infusion of GPI.^7^, ^8^ Ticagrelor and eptifibatide bolus, as compared with ticagrelor and eptifibatide 2‐hour infusion, might maximally inhibit platelet aggregation and reduce the risk of bleeding. However, IPA with ticagrelor and eptifibatide bolus versus 2‐hour infusion has not been studied. We, thus, compared IPA with ticagrelor and eptifibatide bolus versus ticagrelor and eptifibatide bolus with 2‐hour infusion in patients with high‐risk NSTE‐ACS undergoing early PCI. We also compared the incidence of HPR, periprocedural myocardial infarction (PMI), hemoglobin/hematocrit levels post‐PCI, and cardiac events at 1‐year between the 2 groups.

## Methods

### Patient Population

This was a prospective, randomized, single blind study in P2Y_12_‐naïve patients with NSTE‐ACS. Patients were included in the study if they met the following criteria: angina at rest or recurrent angina associated with high‐risk features including elevated cardiac troponin I levels above the upper limit of normal and/or ST‐segment depression of at least 0.1 mV, and a coronary stenosis requiring PCI before randomization. Patients with the following criteria were excluded from the study: upstream use of P2Y_12_, inhibitors, or GPI; left main coronary artery stenosis; cardiogenic shock; thrombocytopenia with platelet count <100 000; severe hepatic impairment; surgery less than 4 weeks; concomitant therapy with strong cytochrome P‐450 3A inhibitors; and a need for oral anticoagulant therapy. The institutional review board approved the study and written informed consent was obtained from all patients.

### Study Design and Randomization

**Figure 1:**
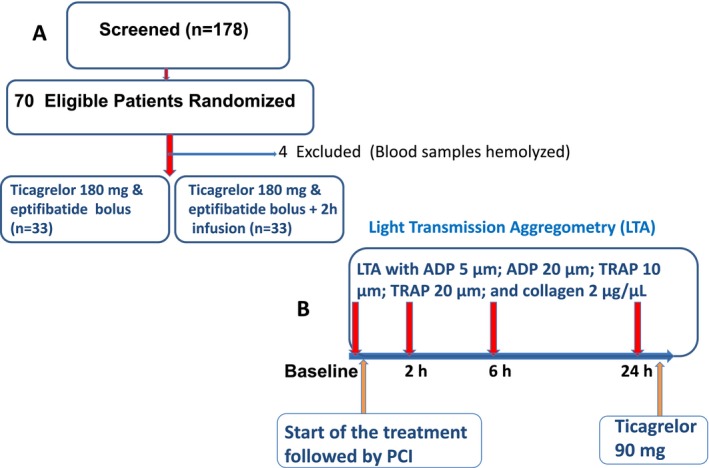
Patient flow and study design. A, demonstrates the number of patients screened and randomized to 2 groups; (B) shows the study design and sampling schedule. Light transmission aggregometry (LTA) was performed at baseline (before administration of ticagrelor and eptifibatide bolus or 2‐hour infusion) and at 2, 6, and 24 hours using ADP, thrombin receptor activating peptide (TRAP), and collagen after the administration of the study drug. Ticagrelor and eptifibatide bolus or infusion were administrated just before percutaneous coronary intervention (PCI).

The study design is shown in Figure 1. Eligible patients who were seen in the emergency room or admitted to the hospital with NSTE‐ACS underwent cardiac catheterization on the same day. After performing cardiac catheterization, eligible patients were randomized if there was a stenosis in the culprit vessel requiring PCI. Randomization was computer generated and was placed in sealed envelopes. Intravenous heparin (60 units/kg) or bivalirudin was administered to the eligible patients before PCI. Patients were randomized 1:1 to group 1 versus group 2. The baseline blood sample was withdrawn for light transmission aggregometry (LTA), and then the study medications (ticagrelor and eptifibatide bolus or 2‐hour infusion) were administered just before PCI. In group 1, ticagrelor (180 mg) and eptifibatide bolus (2 intravenous boluses were administered 10 minutes apart at a dose of 180 μg/kg; the first bolus was administered just before PCI). In group 2, ticagrelor (180 mg) and an eptifibatide bolus 180 μg/kg and a 2‐μg/kg per minute infusion for 2 hours were administered before PCI followed by a second eptifibatide bolus 180 μg/kg 10 minutes later. The maintenance dose of ticagrelor (90 mg twice a day) was started after drawing the last blood sample for PD study at 24 hours. Blood samples for troponin assay were collected at baseline and at 8 and 24 hours post‐PCI.

### Platelet Function Testing Using LTA

The design of the study is demonstrated in Figure 1. After randomization, blood samples were collected at baseline (before administration of ticagrelor and eptifibatide) and at 2, 6, and 24 hours from the time of administration of eptifibatide and ticagrelor.

LTA was performed as previously reported.^9^ Briefly, blood samples were collected in 3.8% sodium citrate tubes. Platelet aggregation was tested using the turbidimetric method in a 2‐channel aggregometer (Chronlog Optical Aggregometer [model 490‐4D]; Chrono‐log Corporation, Havertown, PA). The following agonists were used for platelet stimulation: ADP (5 and 20 μmol/L); thrombin receptor‐activating peptide (TRAP; 10 and 20 μmol/L); and collagen 2 μg/μL. Briefly, platelet‐rich plasma was stimulated by adding an agonist to the cuvette in the aggregometer, and platelet aggregation was recorded as aggregation curves after the addition of each agonist for 6 minutes. The maximal extent of aggregation was expressed as the percent change in light transmittance from the baseline. Each measurement was performed in duplicate, and the average of the 2 measurements was recorded. Percent IPA (%IPA) was calculated as follows: %IPA=100%×(PAt−PAb)/PAb, where PAb is platelet aggregation at baseline, and PAt is platelet aggregation after treatment with ticagrelor and eptifibatide.

### Study End Points and Definitions

The primary end point of the study was IPA stimulated with ADP 20 μmol/L at 2 hours. The secondary end points were as follows: HPR was defined as platelet aggregation >46% or >59% in response to ADP 5 μmol/L or ADP 20 μmol/L, respectively, as previously reported^10^; PMI was defined as an increase in troponin I values >5×99th percentile the upper limit of normal in patients with normal baseline value on admission, or a rise of troponin I values >20% after PCI if the baseline value was elevated^11^; the hemoglobin levels after PCI as compared with baseline in each group; and bleeding complication was classified according to the Bleeding Academic Research Consortium, as previously reported.^12^ The composite of major adverse events (death, myocardial infarction, stent thrombosis, and revascularization) was assessed at follow‐up.

### Clinical Follow‐up

Post‐PCI, patients were scheduled for follow‐up at 1 month and every 6 months thereafter. Major adverse cardiovascular events, including myocardial infarction, stent thrombosis, and target vessel revascularization, were recorded during follow‐up.

### Sample‐Size Calculation and Statistical Analysis

The sample size of the study was determined based on noninferiority analysis that IPA after treatment with ticagrelor and eptifibatide bolus (group 1) stimulated with ADP 20 μmol/L would be noninferior to that of ticagrelor and eptifibatide bolus with 2‐hour infusion (group 2). Because there are no previous data on IPA with ticagrelor and eptifibatide bolus or infusion, we estimated that IPA with ADP 20 μmol/L would be 88% in group 1 and 95% in group 2 at 2 hours, as previously reported.^2^ Assuming a noninferiority margin of 10%,^2^ 66 patients were required to meet the primary end point with a power of 80%. Considering a 5% dropout rate, we randomized 70 patients equally distributed between the 2 groups. We used a 1‐sided *t* test to calculate the noninferiority *P* value.

Continuous variables are shown as mean±SD and were compared by using the unpaired Student *t* test, and the chi‐square test was used to compare categorical variable between the groups. An ANCOVA with a general linear model was used to compare IPA levels between the groups, which were adjusted for baseline value of platelet aggregation, as previously reported.^13^ IPA levels at 2‐, 6‐, and 24‐hour time points within the groups were compared using one‐way ANOVA with Bonferroni correction for multiple testing. Statistical analysis was performed using SPSS software (version 22.0; SPSS Inc, Chicago, IL).

## Results

### Patient Characteristics

Between May 2014 and January 2015, 178 patients with NSTE‐ACS were screened; of these, 70 who met inclusion and exclusion criteria were enrolled in the study and randomized equally to 2 groups: group 1 (ticagrelor+eptifibatide bolus=35 patients) and group 2 (ticagrelor+eptifibatide bolus with 2‐hour infusion=35 patients). In 4 patients, 2 patients in each group, PD analysis could not be performed because blood samples hemolyzed. Therefore, PD analysis was performed in 66 patients (ticagrelor+eptifibatide bolus=33 patients; group 1) and (ticagrelor+eptifibatide bolus plus 2 infusion=33 patients; group 2). The baseline characteristics of the patients were similar between the groups (Table 1). There were no significant differences between the groups with respect to positive troponin or ST‐segment depression (Table 1).

**Table 1:** Demographic and Baseline Characteristics of Patients

	Ticagrelor+Eptifibatide Bolus (n=35) Group 1	Ticagrelor+Eptifibatide Bolus and Infusion (n=35) Group 2	*P* Value
Age, y	63±9	64±14	0.86
Sex, male, n (%)	18 (51)	21 (60)	0.56
Diabetes mellitus, n (%)	6 (17)	8 (23)	0.29
Hypertension, n (%)	23 (66)	26 (74)	0.46
Smoking, n (%)	13 (37)	12 (34)	0.62
Hyperlipidemia, n (%)	17 (48)	20 (57)	0.56
Peripheral vascular disease, n (%)	11 (31)	9 (26)	0.78
Chronic renal failure	3 (8)	4 (11)	0.85
Previous CABG	6 (17)	8 (23)	0.52
Cardiac troponin I level ≥0.04 μg/mL, n (%)	25 (71)	23 (66)	0.12
ST‐segment depression on admission, n (%)	23 (66)	27 (77)	0.39

CABG indicates coronary artery bypass graft.

### Procedural Characteristics of Patients

The procedural characteristics of the patients are displayed in Table 2. There were no significant differences between group 1 (ticagrelor and eptifibatide bolus) and group 2 (ticagrelor and eptifibatide bolus with 2‐hour infusion) with respect to stent diameter, stent length, number of stents, postdilation balloon diameter, and postdilation balloon pressure. PCI was successfully performed in all, except in 1 patient randomized to group 2, in whom the lesion could not be crossed with a guidewire because of vessel tortuosity. The median times from hospital admission to PCI were not significantly different between group 1 versus group 2 (7 versus 6 hours, respectively; Table 2).

**Table 2:** Procedural Characteristics of Patients

	Ticagrelor+Eptifibatide Bolus (n=35) Group 1	Ticagrelor+Eptifibatide Bolus and Infusion (n=35) Group 2	*P* Value
Coronary lesion			
Left main	0	1	
Left anterior descending, n	14	11	
Left circumflex, n	10	12	
Right, n	11	11	
Stent diameter, mm	3.12±0.39	3.13±0.49	0.93
Total stent length, mm	26.9±15.2	25.1±11.5	0.63
No. of stents	1.3±0.60	1.2±0.48	0.47
Drug‐eluting stents, %	94	91	0.81
Postdilation balloon diameter, mm	3.5±0.67	3.5±0.65	0.47
Postdilation balloon length, mm	14.3±3.1	13.2±2.3	0.10
Postdilation inflation pressure, atm	17.1±1.74	17.03±1.78	0.87
Heparin, %	74	78	0.82
Bivalirudin, %	26	22	0.74
PCI, %	100	97	0.85
Median time to PCI, IQR (h)	7 (5.25–9.00)	6 (4–8)	0.46

IQR indicates interquartile range; PCI, percutaneous intervention.

### Assessment of Platelet Aggregation by LTA

**Figure 2:**
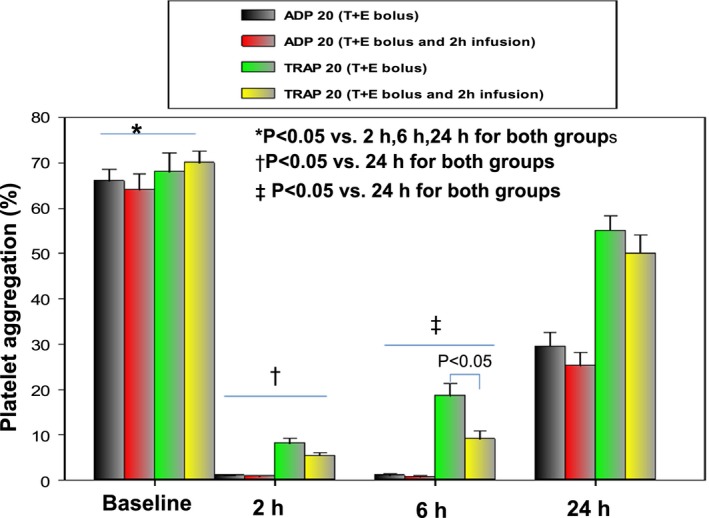
Platelet aggregation (PA) by light transmission aggregometry (LTA). At baseline and at 2, 6, and 24 hours after treatment, gray bars represent PA with ADP 20 μmol/L in the ticagrelor and eptifibatide group (group 1, T+E); red bars represent PA with ADP 20 μmol/L in the ticagrelor and eptifibatide bolus with 2‐hour infusion group (group 2, T+E bolus and 2‐hour infusion); green bars, PA with thrombin receptor‐activating peptide (TRAP) 20 μmol/L in the ticagrelor and eptifibatide group (group 1, T+E); and yellow bars, PA with TRAP 20 μmol/L in the ticagrelor and eptifibatide bolus with 2‐hour infusion group (group 2, T+E bolus and 2‐hour infusion). PA with ADP 20 μmol/L or TRAP 20 μmol/L was significantly higher at baseline than that at 2, 6, and 24 hours. PA with ADP or TRAP was significantly lower at 2 and 6 hours than that at 24 hours.

Ticagrelor strongly inhibits the P2Y_12_ receptor, and its efficacy was tested by ADP. Eptifibatide strongly inhibits GP IIb/IIIa receptors, and its efficacy was assessed by TRAP. As shown in Figure 2, the baseline values of platelet aggregation (PA) with ADP 20 μmol/L and TRAP 20 μmol/L were not significantly different between the groups. In group 1, after administration of ticagrelor and eptifibatide bolus (T+E bolus), and in group 2 after ticagrelor and eptifibatide bolus with 2‐hour infusion (T+E bolus with 2‐hours infusion), ADP‐ and TRAP‐induced PA significantly decreased at 2, 6, and 24 hours as compared with their baseline values. Both ADP‐ and TRAP‐induced PA values were significantly lower at 2 and 6 hours as compared with 24 hours. TRAP (20 μmol/L)‐induced PA was significantly higher in group 1 than in group 2 at 6 hours.

### Assessment of Inhibition of PA by LTA

The primary end point of the study, IPA levels at 2 hours after platelet stimulation with ADP 20 μmol/L, were similar in both groups (99.59±0.43% in group 1 versus 99.88±1.0% in group 2; mean difference=0.29%; upper bound of 95% CI, 0.71%; *P*<0.001 for noninferiority).

**Figure 3:**
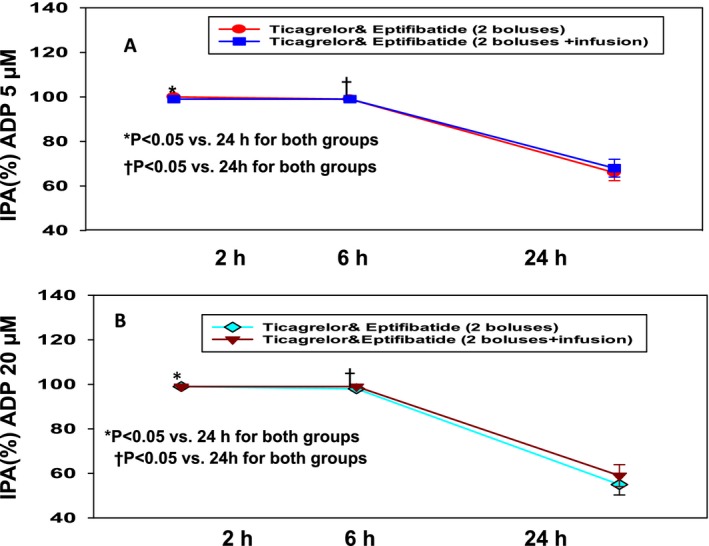
Inhibition of platelet aggregation (IPA) with ADP. A, IPA stimulated with ADP 5 μmol/L after treatment with ticagrelor and eptifibatide (T+E bolus) vs ticagrelor and eptifibatide bolus with 2‐hour infusion (T+E bolus and 2‐hour infusion). B, IPA stimulated with ADP 20 μmol/L after treatment with ticagrelor and eptifibatide (T+E) bolus vs ticagrelor and eptifibatide bolus with 2‐hour infusion (T+E bolus and 2‐hour infusion). In both groups (A and B), IPA levels were significantly higher at 2 and 6 hours than 24 hours.

IPA levels after stimulation with ADP 5 μmol/L and ADP 20 μmol/L were not significantly different between the groups at 2 and 6 hours (Figure 3A and 3B; Table 3). IPA levels with ADP 5 μmol/L and ADP 20 μmol/L in groups 1 and 2 were significantly lower at 24 hours than at 2 and 6 hours (Figure 3A and 3B; Table 3).

**Table 3:** Inhibition of Platelet Reactivity

	Ticagrelor+Eptifibatide Bolus (n=33) Group 1 (%)	Ticagrelor+Eptifibatide Bolus and Infusion (n=33) Group 2 (%)	*P* Value
%IPA (ADP 20 μmol/L)			
2 h	99.59±0.43	99.88±1.0	0.18
6 h	98.4±4.4	99.31±2.9	0.40
24 h	55.7±23	59.4±27	0.45
%IPA (ADP 5 μmol/L)			
2 h	99.9±0.90	99.9±0.25	0.98
6 h	99.1±2.4	99.5±1.9	0.84
24 h	66.27±17.6	68.4±22.3	0.74
%IPA (TRAP 20 μmol/L)			
2 h	88.9±8.1	92±5.5	0.10
6 h	74. 4±17.5	87.4±13.5	0.004
24 h	21.12±19.5	27.8±34.2	0.78
%IPA (TRAP 10 μmol/L)			
2 h	96.6±4.9	97.5±4.6	0.53
6 h	86.4±16.4	93.9±9.7	0.071
24 h	35±27	32±44	0.074
%IPA (collagen 2 μmol/L per μL)			
2 h	99.7±0.72	99.6±0.96	0.60
6 h	97.5±7.9	98.5±4.2	0.60
24 h	61.6±44	68±45	0.60

IPA indicates inhibition of platelet reactivity; TRAP, thrombin receptor‐activating peptide.

**Figure 4:**
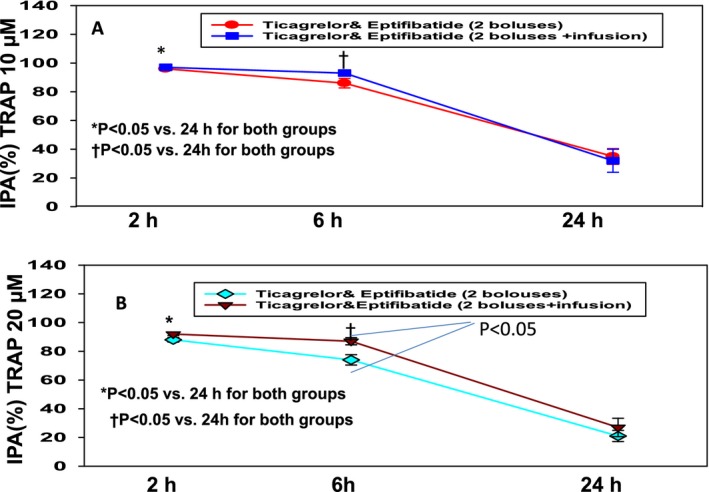
Inhibition of platelet aggregation (IPA) with thrombin receptor activating peptide (TRAP). A, IPA with TRAP 10 μmol/L after treatment with ticagrelor and eptifibatide (T+E) bolus vs ticagrelor and eptifibatide bolus with 2‐hour infusion (T+E bolus and 2‐hour infusion). B, IPA with TRAP 20 μmol/L after treatment with ticagrelor and eptifibatide (T+E) bolus vs ticagrelor and eptifibatide bolus with 2‐hour infusion (T+E bolus and 2‐hour infusion). In both (A and B), IPA levels were significantly higher at 2 and 6 hours than 24 hours. IPA level was significantly higher with T+E bolus and 2‐hour infusion than with T+E at 6 hours.

IPA levels after stimulation with TRAP 10 μmol/L and TRAP 20 μmol/L were not significantly different between the groups at 2 and 6 hours (Figure 4A and 4B; Table 3). IPA levels with TRAP 10 μmol/L and TRAP 20 μmol/L in groups 1 and 2 were significantly lower at 24 hours as compared with those at 2 and 6 hours (Figure 4A and 4B; Table 3). IPA level with TRAP 20 μmol/L was significantly higher in group 2 as compared with that in group 1 at 6 hours (87.4±14% versus 74±17%; *P*<0.01, respectively; Figure 4B).

IPA levels after stimulation with collagen 2 μmol/L/μL were not significantly different between the groups at 2 and 6 hours (Table 3). IPA levels with collagen 2 μmol/L/μL in groups 1 and 2 were significantly lower at 24 hours than at 2 and 6 hours (Table 3).

### High On‐Treatment Platelet Reactivity

**Figure 5:**
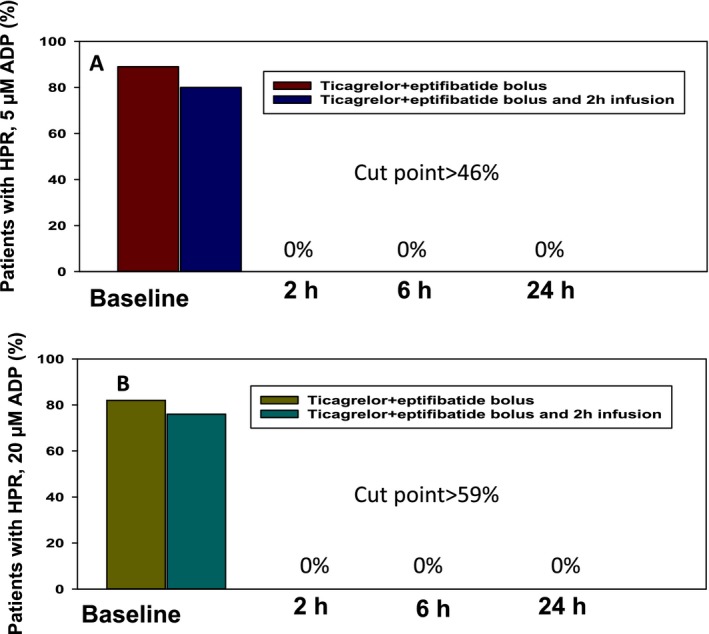
Percentages of high on‐treatment platelet reactivity (HPR). A, percentages of HPR with ADP 5 μmol/L using the cut point >46%. At baseline, percentages of HPR were 89% and 79% (*P*=0.46). At 2, 6, and 24 hours, percentages of HPR were 0, indicating that ticagrelor and eptifibatide bolus or infusion completely blocked platelet reactivity stimulated with ADP 5 μmol/L. B, percentages of HPR with ADP 20 μmol/L using the cut point >59%. At baseline, percentages of HPR were 82% and 76% (*P*=0.87). At 2, 6, and 24 hours, percentages of HPR were 0, indicating that ticagrelor and eptifibatide bolus or infusion completely blocked platelet reactivity stimulated with ADP 20 μmol/L.

As shown in Figure 5A, the percentages of HPR stimulated with ADP 5 μmol/L were 89% and 79% before treatment in groups 1 and 2, respectively (*P*=0.46). HPR with ADP 5 μmol/L dropped to 0 at 2, 6, and 24 hours after treatment in both groups.

As shown in Figure 5B, the percentages of HPR stimulated with ADP 20 μmol/L were 82% and 76% before treatment in groups 1 and 2, respectively (*P*=0.87). HPR with ADP 20 μmol/L dropped to 0 at 2, 6, and 24 hours after treatment in both groups.

### Outcomes

Details of in‐hospital and follow‐up outcomes are shown in Table 4. One patient in group 2 developed gastrointestinal bleeding post‐PCI and required a blood transfusion. Incidence of PMI was not significantly different between the groups. Post‐PCI, hemoglobin level was significantly lower in group 2 as compared with baseline; however, no source of bleeding was identified. In group 1, 1 patient developed NSTE‐ACS as a result of in‐stent restenosis at 6 months. In group 2, there were 2 events; 1 patient died of progressive heart failure, and another patient developed in‐stent restenosis and required target lesion revascularization at 4 and 7 months, respectively.

**Table 4:** Outcomes

	Ticagrelor+Eptifibatide Bolus (n=35) Group 1	Ticagrelor+Eptifibatide Bolus and Infusion (n=35) Group 2	*P* Value
In‐hospital events			
Periprocedural myonecrosis, n (%) (>5×99th percentiles or >20% increase in troponin levels)	9 (26)	7 (20)	0.57
Baseline hemoglobin, g/dL	13.62±1.54	13.35±1.8	0.84
Postprocedure hemoglobin, g/dL	13.04±1.55	12.38±1.80^a^	0.22
Bleeding (academic Research Consortium 3b)	0	1	
Follow‐up events			
Duration of follow‐up, month	10±4.4	11±3.7	
Death	0	1	
Stroke	0	0	
TLR		1	
NSTEMI	1		
Major adverse cardiovascular events, n	1	2	

a *P*<0.05 compared with baseline hemoglobin. NSTEMI indicates non‐ST‐elevation myocardial infarction; TLR, target lesion revascularization.

## Discussion

To the best of our knowledge, the present study is the first to investigate the effects of ticagrelor and eptifibatide bolus versus ticagrelor and eptifibatide bolus with 2‐hour infusion in patients with high‐risk NSTE‐ACS undergoing early PCI. The results of our study are summarized as follows:

1. In P2Y_12_‐naïve patients with high‐risk NSTE‐ACS undergoing early PCI, IPA with ticagrelor and eptifibatide bolus was maximal at 2 hours, the time frame in which most patients undergo PCI, suggesting that eptifibatide 2‐hour infusion is not needed.
2. Maximal inhibition of PA with ticagrelor and eptifibatide bolus is further supported by fact that platelet reactivity was below the cut points associated with ischemic risk.
3. Simultaneous inhibition of GPI and P2Y_12_ receptors with eptifibatide and ticagrelor, respectively, provided a synergistic effect, such that eptifibatide, a strong TRAP‐induced platelet inhibitor, led to short‐term platelet inhibition during PCI, and ticagrelor, a strong ADP‐induced platelet inhibitor, induced sustained platelet inhibition up to 24 hours.
4. Ticagrelor and eptifibatide bolus versus 2‐hour infusion did not drop the post‐PCI hemoglobin level and may reduce bleeding complications.
5. PMI and cardiac events at 1 year were not significantly different between the groups.

Kim et al^2^ reported that IPA with ticagrelor alone was inferior to tirofiban infusion at 2 hours in patients with NSTE‐ACS undergoing PCI. Angiolillo et al^3^ showed that in P2Y_12_‐naïve patients with unstable angina, the rate of HPR with ticagrelor was still high at 2 hours. Likewise, Valgimigli et al^14^ showed that IPA with prasugrel and tirofiban bolus or 2‐hour infusion was significantly higher than prasugrel alone in patients with ST‐segment elevation myocardial infarction. Furthermore, the ATLANTIC trial^15^ demonstrated that the prehospital administration of ticagrelor did not improve pre‐PCI coronary reperfusion because maximal IPA with ticagrelor did not occur until 1 hour post‐PCI. Taken together, the above series indicate that even potent P2Y12 inhibitors (ie, prasugrel or ticagrelor) when administrated before PCI, do not achieve maximal platelet inhibition until the first 2 hours, the time frame in which most patients undergo PCI. Therefore, GPI is still needed to achieve maximal platelet inhibition quickly.

Notably, in the present study, there was no instance of HPR with ticagrelor and eptifibatide bolus. Mangiacapra et al^16^ reported that HPR was an independent predictor of PMI after PCI. Likewise, Valgimgli et al^17^ reported that treatment with tirofiban, in patients who had HPR with clopidogrel, resulted in a 40% reduction in the incidence of PMI compared with clopidogrel. Although it has been assumed that the use of potent P2Y_12_ inhibitors would overcome the problem of HPR, recent data^4^ showed that HPR was still observed in a significant number of patients (25.2%) with prasugrel, a potent P2Y_12_ inhibitor.

We demonstrated that the median time from admission to PCI was between 6 and 7 hours. A number of randomized trials^18^, ^19^ showed that early or immediate PCI reduced the event rates in high‐risk patients. Given the absence of robust data on the pretreatment strategy and the fact that the interval between admission and PCI has now shortened with modern practice, our study supports the concept of synergistic antiplatelet therapy with ticagrelor and eptifibatide bolus in high‐risk patients with NSTE‐ACS patients undergoing early PCI.

### Clinical Implications

We showed that ticagrelor and eptifibatide bolus maximally inhibited PA and there was no instance of HPR, indicating that platelet reactivity was below the cut points associated with ischemic risk. Given that a number of studies^16^, ^17^ reported an association between HPR and increased PMI, the absence of HPR with the use of ticagrelor and eptifibatide bolus should reduce such events.

Current guidelines^5^ recommend a GPI infusion for 12 to 18 hours in high‐risk ACS patients. A GPI bolus or 2‐hour infusion with clopidogrel, as compared with a GPI infusion for 12 to 18 hours, decreased bleeding complications with no difference in outcomes.^7^, ^8^ Furthermore, Gurm et al^20^ analyzed 21 296 patients; of these, 4511 were treated with eptifibatide bolus at the time of PCI and 16 785 received standard therapy (bolus plus infusion). They showed that patients receiving eptifibatide bolus only had significantly lower rates of bleeding events and blood transfusion with no difference in adverse events. Likewise, Kini et al^7^ demonstrated that GPI bolus, compared with GPI bolus with standard infusion in 2699 patients undergoing PCI, reduced bleeding, duration of hospitalization, and cost with no difference in outcome. Furthermore, in the present study, there was no hemoglobin drop with ticagrelor and eptifibatide bolus in patients randomized to ticagrelor and eptifibatide bolus. In this respect, a randomized trial is warranted to investigate outcomes of high‐risk patients with NSTE‐ACS undergoing early PCI. Furthermore, randomized trials are warranted to investigate the safety and costs of ticagrelor and eptifibatide bolus versus cangrelor^21^ (a fast‐acting intravenous P2Y_12_ inhibitor) and ticagrelor in high‐risk NSTE‐ACS patients undergoing early PCI.

### Limitations of the Study

In the present study, we used LTA to analyze platelet function. The most important advantage of LTA is that there is good correlation between the results and adverse events.^22^ There are several limitations of the study. First, a small patient population and therefore a small number of outcome events limit this study. Second, we did not compare the PD effect of ticagrelor and eptifibatide bolus or 2‐hour infusion with ticagrelor+unfractionated heparin/bivalirudin. In this respect, future studies are needed to compare the PD effect of ticagrelor and eptifibatide bolus with ticagrelor and unfractionated heparin/bivalirudin. Third, because clopidogrel and eptifibatide 18‐ versus 2‐hour infusion increased bleeding,^8^ we did not randomize patients to ticagrelor and eptifibatide 18‐ versus 2‐hour infusion. Fourth, because it has been demonstrated^23^ that the vasodilator‐stimulated phosphoprotein assay had minor differences as compared with the LTA measurements, we did not perform the vasodilator‐stimulated phosphoprotein assay to measure platelet reactivity index. Instead, multiple platelet function tests were performed in duplicates.

## Conclusions

This is the first study to demonstrate that in P2Y_12_‐naïve patients with high‐risk NSTE‐ACS undergoing early PCI, ticagrelor and eptifibatide bolus only, as compared with ticagrelor and eptifibatide bolus with 2‐hour infusion, maximally inhibited PA, which is further supported by platelet reactivity below the cut points of ischemic risk. This indicates that eptifibatide 2‐hour infusion is not needed.

## Sources of Funding

This study was supported by a grant from AstraZeneca. The collection, management, analysis, and interpretation of the data were performed by the UAB Cardiovascular Clinical trial Unite, Dr Marian, and Dr Leesar.

## Disclosures

Dr Leesar received an institution Grant from AstraZenca.
